# A pure dermal sling for implant reconstruction after mastectomy in the generous breast

**DOI:** 10.1308/003588412X13373405385214g

**Published:** 2012-07

**Authors:** P Sarmah, N Abbott, R Bright-Thomas

**Affiliations:** Worcestershire Acute Hospitals NHS TrustUK

## BACKGROUND

Skin reducing mastectomy is a useful technique in immediate implant-based reconstruction. The implant is usually covered by muscle and a dermal flap.^1^ We describe a modification to this technique for generous breasts involving the creation of a pure dermal sling.

**Figure 1:**
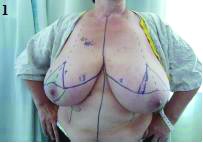
Pre-operative marking for Wise pattern incisions

## TECHNIQUE

A skin reducing mastectomy is performed via Wise pattern incisions, retaining an extensive inferior dermal sling of de-epithelialised tissue. A sizer is used to ensure the skin envelope meets the inframammary fold before an anatomical implant is placed on the pectoralis major. Complete coverage is achieved with the inferior dermal sling, which is sutured to the pectoralis superiorly. The superior skin can then be draped to the inframammary fold and sutured in the usual manner.

## DISCUSSION

The benefits of a pure dermal sling include complete coverage of the implant and the preservation of an intact pectoralis major. This technique has been trialled successfully in two patients with generous ptotic breasts.

**Figure 2:**
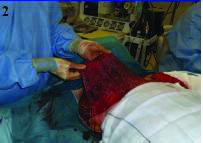
Pure dermal sling

**Figure 3:**
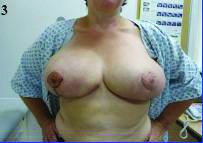
After reconstruction using pure dermal sling

**Figure 4:**
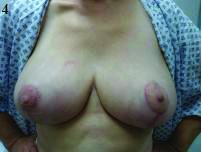
Results at four-month follow-up appointment
